# Statistical analysis and decision making in cancer screening

**DOI:** 10.1007/s10654-018-0406-8

**Published:** 2018-05-12

**Authors:** Dik Habbema

**Affiliations:** Department of Public Health, Erasmus MC University Medical Center, Rotterdam, The Netherlands

Randomized controlled trials are the primary source of evidence for assessing the effectiveness of cancer screening. Thus far, trial data have mainly been analyzed using relative risk estimates or proportional hazard models [1]. Proportional hazard models assume that screening results in a time-independent reduction in cancer mortality. Hanley, Liu and coworkers have developed a model with a time-dependent mortality reduction, see elsewhere in this issue of the journal [2, 3]. The model assumes that the reduction in mortality from the target cancer appears after a delay following a screen, and eventually disappears. Mortality reductions from subsequent screening rounds are superimposed. The resulting function has a bathtub form, and is determined by two parameters: the time between a screen and maximum relative mortality reduction, and the value of the maximum relative mortality reduction [2]. The authors have applied their method to data from prostate cancer [1], lung cancer [3], colorectal cancer [3] and breast cancer [2], using excellent graphical illustrations (Figures 3 and 4 in [3]).

The Hanley–Liu model is more realistic than the proportional hazard model. In practice, discriminating between the two models can be difficult. Designers of screening trials are aware of the bathtub dynamics of mortality reduction. They mitigate the influence of the initial phase of (near) absence of reduction by excluding persons with an already established diagnosis of the target cancer. A good compromise follow-up duration is the crux for dealing with the tapering off phase at the end. Follow-up should neither be too short when mortality reduction is still increasing nor too long with much noise from deaths which could not have been prevented by screening anyhow. With these choices, most cancer deaths in screening trials will occur in the bottom part of the bathtub, where the constant mortality reduction of the proportional hazard model is a good approximation to the Hanley–Liu model. And indeed, it proved not to be possible to discriminate between the two models in the analysis of the Danish breast cancer data [2]. The scatter of the time-dependent relative mortality dots in Figures 3 and 4 in [3] suggests that this might also be the case for the lung cancer and colorectal cancer analyses. This lack of discrimination with more complex models might be a reason why the simple proportional hazard model has persisted as the model of choice for statistical analysis of trial data.

The time-dependent mortality reduction curve of the Hanley–Liu model allows us to reflect on trial design issues like screening interval, follow-up time and power analysis.

In order to provide maximal information, the interval between subsequent screenings should be sufficiently long to provide information about the whole trajectory of the bathtub mortality reduction curve. A trial with 3-year intervals will be more informative than a trial with 1-year intervals.

Contrary to the proportional hazard model, duration of follow-up is not crucial for the Hanley–Liu model. While mortality after long follow-up is a source of random noise in the proportional hazard model, it is informative in the Hanley–Liu model for estimating the dynamics of the mortality reduction.

The high costs of screening trials strongly depend on their size. Because of the use of the time-dimension of the mortality data, power calculations will undoubtedly lead to a smaller sample size for the Hanley–Liu model than for the proportional hazard model.

Hanley and Liu note that use of their model is hindered by sparse data. This problem would even become worse when important determinants like age at first invitation and rank of the screening round would be included in the model [2]. The appeal of Hanley and Liu to screening data owners to collaborate is therefore timely and should be endorsed. In addition, it could be recommended that Lexis diagrams as used by Hanley and Liu, with number of deaths and person years at risk in each cell [2] should routinely be included in reports of screening trial results. The Lexis diagram has an age- and a calendar-time axis, describes how cohorts progress along these axes and constitutes a database for further epidemiologic analysis [4].

Mortality analysis of screening trials usually takes place between 15 and 30 years after start of the trial. During this period, some of the biological and behavioral processes which underlie the mortality effects of cancer screening will have changed. Underlying processes which can change over time include incidence of cancer, the stage distribution of diagnosed cancers in the absence of screening, the stage-specific survival of cancer with current treatment, the sensitivity and specificity of screening tests in different disease stages, compliance to the screening, the characteristics of further diagnostics in case of an abnormal screening test result, and the stage specific survival in screen detected cancers, including precursor lesions. For example participants in the Minnesota trial for colorectal cancer screening were (healthy) volunteers, and since the trial the FOBT has largely been replaced by quantitative immunochemical blood tests and new cancer treatments have become available. The proportional hazards and Hanley–Liu models can both be characterized as modeling the mortality response to a screening stimulus which is delivered in the context of underlying processes. The models have no mechanism for correcting the response for secular changes in the underlying processes. This is a major problem for using the results of a statistical analysis beyond the trial context, for example for guideline development.

Many beneficial and harmful outcomes have to be taken into account when comparing screening policies, including overtreatment, anxiety after positive screening tests and complications from screening, follow-up tests and treatment. See [5] for a table of outcomes for colorectal cancer screening. Only one of the outcomes, mortality, is addressed by the proportional hazards and Hanley–Liu models. Mortality is arguable the most important outcome, as cancer screening without mortality reduction is useless.

The mortality output of the Hanley–Liu model which consists of the curve of relative mortality between screening and control group has to be processed before it can be used in decision making. A switch has to be made from relative to absolute mortality, in order to avoid that high and low cancer incidence situations would be treated the same. Age of death should be taken into account by calculating the expected number of life-years gained when preventing a death. Otherwise, prevented deaths at age 50 and age 90 would be valued the same. Two further possible actions are adjustment for time-preference by putting more weight on nearby compared to far away life-years, and adjustment for quality of life by calculating quality-adjusted life years [6].

The suggestion that the Hanley–Liu model can be used for deriving optimal ages and frequency of screening [2] is rather optimistic in view of the need to correct for secular changes and to weigh many harms and benefits. It might be better to turn to mathematical models which are developed with their use for decision making in mind. These so-called decision analytic models consider demography, epidemiology, natural history, screening tests, treatment and other processes, and aim to integrate available data to estimate the health consequences of alternative screening strategies [7]. By now, decision analytic models have been developed in many fields of medicine. For cancer screening, a large number of model groups collaborate in the Cancer Intervention and Surveillance Modeling Network (CISNET). The models have been described in a standardized way, see https://cisnet.cancer.gov/resources/profiles.html. Decision analytic models are increasingly used for informing screening guidelines development, for example by the United States Preventive Services Task Force [8, 9].

The scientific status of decision analytic models is unclear. While statistical models are developed within the firm context of probability theory and theoretical statistics [3], relevance is the primary concern in the development of decision analytic models. In order to increase their trustworthiness, general recommendations for good research practice in decision analytic modeling have been formulated [7]. For cancer screening, model quality and relevance have been discussed in [10]. The quality and credibility of decision models strongly depends on their performance in reproducing results of screening studies. They are considered most useful in situations where strong primary data are available [10]. For example, parameters of a decision analytic model for colorectal cancer screening could be fitted to the results of three randomized trials [11]. In view of the complexity of decision analytic models, much can be gained from collaboration between modeling groups [12] and from multi-model studies [13].

In conclusion, statistical models and decision analytic models are both important in cancer screening. Statistical models are essential for analysis of trial data. Decision analytic models are used in screening guidelines development. Decision modelers can learn from statistical models for improving the fitting and validation of primary data. Statistical modelers can learn from decision analytic models for improving the usefulness of their models for decision making. Hanley and Liu have improved on existing statistical models. By modeling the time dimension of the mortality reduction they improved the relevance for decision making, especially with regard to the question of optimal screening intervals. Decision analytic modelers should in turn try to learn from the Hanley–Liu model for improving the ways in which they fit their model to primary data.
